# Œdema of the Upper Extremity Following Injuries to the Olecranon Bursæ

**Published:** 1898-11-19

**Authors:** J. Ernest Frazer

**Affiliations:** Formerly Senior House Surgeon Metropolitan Hospital, &c.


### Hospital Clinics and Medical Progress.

### ŒDEMA OF THE UPPER EXTREMITY FOLLOWING INJURIES TO THE OLECRANON BURSÆ.

By J. Ernest Frazer, F.R.C.S., formerly Senior House Surgeon Metropolitan Hospital, &c.

It is not a very uncommon thing to see œdema of the hand and forearm following injuries of the structures over the olecranon process. The condition, which is not a cellulitis, is very chronic and troublesome, though not painful; so far as I am aware, it does not receive any notice in surgical text-books. The patient observes (usually the day after the receipt of the injury) that the back of the hand is œdematous, the swelling first showing itself over, or rather, between, the distal ends of the metacarpal bones, from whence it gradually extends up the forearm, and occasionally beyond the elbow. It is white and soft, easily and deeply pitting on pressure, and not accompanied by fever or pain. Over the olecranon a ragged contused wound will be found, resulting generally, according to the patient, from a "knock on the elbow." Having reached its highest point and become stationary, the swelling remains *in statu quo* for an indefinite time—weeks or months—troubling the patient more by its mechanical interference with the use of the hand than by any pain or tenderness. When this condition is present it will be found that any local treatment by fomentations, dressings, &c., is absolutely useless; the swelling remains the same, and the wound shows very slight tendency to heal. Having found from experience that this was so, and that the bursæ apparently were at fault, it seemed to me that a radical treatment by removal of these structures might be tried, and this I have found invariably successful. There are, usually, at least two bursæ in the neighbourhood of the olecranon, both lying between the superficial fascia and the deeper structures, in the subcutaneous cellular plane. The larger one, the "olecranon bursa" proper, is situated behind the base of the process, and is invariably present, at any rate after childhood; the second and smaller one, which is, if not constant, at least very frequently present, is situated an inch and a half, or more, lower down the forearm, lying partly on the subcutaneous border of the ulna and partly on the deep fascia. The position of these two sacs can be seen and felt in those that are the subjects of gouty bursal deposits. Their removal is easily effected by marking out, with a curved incision, a flap, with its base downwards, formed of skin and superficial fascia; when this is turned back, the subcutaneous cellular layer is exposed. The bursæ generally remain adherent to the deep structures. Everything is now cleared away from the bone and deep fascia till they are left cleanly exposed, then the deep surface of the flap is scraped, and it is sutured back in position; the original wound is included in the incision, or is removed by a separate one, if necessary. The limb is then placed on a moulded anterior splint in the position of natural flexion, and the wound is re-dressed at the end of a week, when there should be no œdema, and the wound should have healed by first intention. I have never been disappointed in either result. Although this treatment proved so easy and so successful, it was not so easy to give a ground for its necessity, or a reason for its success. The onset and clinical course of the disorder certainly points to gravity as having some influence. Evidently there is exudation, arising at the seat of injury, finding its way in the subcutaneous plane to the most dependent point: an identical appearance can be produced in the dead body by slowly injecting water into the subcutaneous tissue when the limb is hanging down. The important point, however, lies in the reason why this fluid apparently remains *in situ*, and is not absorbed. To reach an explanation of this seemed to me at one time to be practically impossible under the theories of the day regarding absorption and exudation. In the last few years, however, initiated by Heidenhain's researches on the subject, a good deal of work has been done, and many new facts and factors have been brought forward bearing on these two processes. In the light of these new facts, I think an explanation—purely a theoretical one—can be found to give a rational basis for treatment. Dr. Starling, in the Arris and Gale Lectures for 1896, stated his views on the matter fully, as foreshadowed by previous papers by him and other workers; papers published since then have borne out these views, with slight modifications and additions, and they are, I think, generally accepted in England.

For those who have not followed this question, I will state shortly the facts that bear on the present subject: (1) Exudation, which takes place through the capillaries, varies, *cæteris paribus*, with the capillary pressure; (2) absorption takes place through the capillaries, and varies inversely as the osmotic proteid pressure of the exudation compared to that of the intra-vascular fluid, in any given capillary area; (3) the percentage of proteids in the exudation varies inversely as the resistance or "thickness" of the capillary walls through which it takes place; this "thickness" is greater in the limbs and connective tissues than in the visceral or serous tissues; (4) the percentage of proteids in the exudation of any set of capillaries varies inversely as the pressure in that set of capillaries, *cæteris paribus*.

From a consideration of these statements it will be seen that a balance can be (and evidently is) kept between exudation from any set of vessels and absorption by them: if exudation increases it becomes thinner in proteids, and is therefore more rapidly absorbed, and *vice versâ*—in other words, the factors causing variation in the rate of exudation provide at the same time for a corresponding variation in the rate of absorption. But these conditions evidently only apply when the processes of absorption and exudation are accomplished by the same set of vessels, or, at any rate, by vessels of the same "thickness," and under the same conditions. If the set of vessels that provided the exudation differed in this respect from those that were set the work of absorption, the relations between the two processes would assuredly break down at once. In this fact, I imagine, lies the explanation for which we have been seeking. The second bursa rapidly enlarges, and ruptures in the subcutaneous plane. I have seen this bursa fully distended on turning back the skin a few hours after the injury to the olecranon, but I have not seen it when doing so after the œdema has set in. As the open wound is not over the second sac, the exudation, when set free in the cellular tissue, does not drain externally, but finds its way through that tissue to the most dependent spot. If now we assume, as I think we are justified in doing, that the vessels providing the exudation in the bursa are considerably "thinner" than the "thick" vessels of the limb outside, it is evident that absorption cannot go on *pari passu* with exudation, so that the fluid must collect, thus bringing an increasing absorption area into play, until the quantity absorbed equals that exuded, when, of course, the amount of fluid remains stationary. If this explanation is correct, it still does not explain why the exudation should continue for so long a time; yet the rapid disappearance of œdema on removal of the bursa certainly points to removal of a previously existing source of exudation. Whatever the cause of this may be, it need not detain us here; it is probably due to some irritation, chemical or otherwise, from the injured spot close by, and it is not inconceivable that this irritation might still further reduce the "thickness" of the vessel walls, and thus increase the thickness of their exudation. When the bursæ have been removed, absorption can go on at its own speed; the fluid tends to become more concentrated as it is absorbed, but this is neutralised by the proteids being used up by the tissue cells, or disposed of otherwise.

I have once or twice, when operating, found that the olecranon was fractured through its base, the fragments being in close opposition, and moveable with difficulty. The possibility of this complication being present should be borne in mind, for, although it does not affect the treatment of the bursæ, it considerably modifies the after-course of the case, and may introduce after-effects otherwise difficult to explain.

